# *Mycobacterium tuberculosis* Population in Northwestern Russia: An Update from Russian-EU/Latvian Border Region

**DOI:** 10.1371/journal.pone.0041318

**Published:** 2012-07-23

**Authors:** Igor Mokrousov, Anna Vyazovaya, Tatiana Otten, Viacheslav Zhuravlev, Elena Pavlova, Larisa Tarashkevich, Valery Krishevich, Boris Vishnevsky, Olga Narvskaya

**Affiliations:** 1 Laboratory of Molecular Microbiology, St. Petersburg Pasteur Institute, St. Petersburg, Russia; 2 Laboratory of Microbiology, Research Institute of Phthisiopulmonology, St. Petersburg, Russia; 3 Laboratory of Molecular Genetic Methods, Research Institute of Phthisiopulmonology, St. Petersburg, Russia; 4 Bacteriology Laboratory, Pskov Oblast Anti-tuberculosis dispensary, Pskov, Russia; The University of Hong Kong, China

## Abstract

This study aimed to characterize the population structure of *Mycobacterium tuberculosis* in Pskov oblast in northwestern Russia, to view it in the geographical context, to compare drug resistance properties across major genetic families. Ninety *M. tuberculosis* strains from tuberculosis (TB) patients, permanent residents in Pskov oblast were subjected to LAM-specific IS*6110*-PCR and spoligotyping, followed by comparison with SITVITWEB and MIRU-VNTRplus databases. The Beijing genotype (n = 40) was found the most prevalent followed by LAM (n = 18), T (n = 13), Haarlem (n = 10), Ural (n = 5), and Manu2 (n = 1); the family status remained unknown for 3 isolates. The high rate of Beijing genotype and prevalence of LAM family are similar to those in the other Russian settings. A feature specific for *M. tuberculosis* population in Pskov is a relatively higher rate of Haarlem and T types. Beijing strains were further typed with 12-MIRU (followed by comparison with proprietary global database) and 3 hypervariable loci QUB-3232, VNTR-3820, VNTR-4120. The 12-MIRU typing differentiated 40 Beijing strains into 14 types (HGI = 0.82) while two largest types were M2 (223325153533) prevalent throughout former USSR and M11 (223325173533) prevalent in Russia and East Asia. The use of 3 hypervariable loci increased a discrimination of the Beijing strains (18 profiles, HGI = 0.89). Both major families Beijing and LAM had similar rate of MDR strains (62.5 and 55.6%, respectively) that was significantly higher than in other strains (21.9%; *P* = 0.001 and 0.03, respectively). The *rpoB531* mutations were more frequently found in Beijing strains while LAM drug resistant strains mainly harbored *rpoB516* and *inhA* −15 mutations. Taken together with a high rate of multidrug resistance among Beijing strains from new TB cases (79.3% versus 44.4% in LAM), these findings suggest the critical impact of the Beijing genotype on the current situation with MDR-TB in the Pskov region in northwestern Russia.

## Introduction

The permanent development of the novel and fine-tuning of the existing molecular approaches permitted a spatio-temporal surveillance of the circulating clones of *Mycobacterium tuberculosis* at global and within-country levels. Association of certain genetic variants of *M. tuberculosis* in Russia with pathogenic properties has been demonstrated in some settings, especially for the Beijing genotype strains although other predominant genotypes deserve no less attention as well. To date, the population of *M. tuberculosis* strains circulating in Russia has been well characterized by different typing methods, although the use of different methodologies across different studies makes it difficult to draw comparable conclusions. Nonetheless, information gained by IS*6110*-RFLP, 12-MIRU-VNTR and spoligotyping is available in many settings within Russia and in the neighboring countries. Most of the studies carried out by us and others on the vast area of the northwestern Russia focused mainly on St. Petersburg [1], [2], Karelia [3], Archangel [4], Murmansk [5] while other provinces received no attention at all or much smaller attention due to inherent limitation of the small sampling per location (e.g., [6]).

The increased human migration results also in active cross-border dissemination of strains hence interest to target such borderline locations. Pskov oblast (670,000 population, 55,400 sq. km surface) is located in northwestern Russia on the Russian-EU/Latvian border. The tuberculosis (TB) incidence here was reported 94.9/100,000 in 2008, 86.7/100,000 in 2009, 82.3/100,000 in 2010 70.8 in 2011 and thus shows a clear trend to decrease; TB prevalence was reported 159.5/100,000 in 2011. At the same time, the rate of drug resistant, and especially, multi-drug resistant TB (MDR-TB) is increasing: MDR-TB was diagnosed in 16.7% of newly diagnosed patients with pulmonary TB in 2011 (V. Krishevich, unpublished data).

The interest of the present study was to characterize the population structure of *M. tuberculosis* in the Pskov oblast in northwestern Russia, to view it in the wide geographical context, and to compare distribution of phenotypic and genotypic drug resistance characteristics across major spoligotype-defined families. Additional analyses/markers were applied to the largest and clinically and epidemiologically important Beijing genotype: (a) 12-MIRU-VNTR loci typing followed by global and regional comparison, (b) use of three hypervariable (HV) VNTR loci to increase a discrimination.

## Materials and Methods

According to the ethics boards of St. Petersburg Institute of Phthisiopulmonology and St. Petersburg Pasteur Institute, this research does not require ethical approval. The DNA samples were without any personal information about the patients in particular without any ID by name, address, i.e. anonymous samples.

### Study sample

The 90 studied strains were selected randomly among strains isolated in bacteriology laboratory of Pskov Anti-tuberculosis dispensary from September, 2008 to March, 2009. They included 65 and 25 strains from newly-diagnosed and previously-treated patients, respectively. In particular, 65 strains from newly-diagnosed patients represented 30.6% of all culture-positive new TB cases diagnosed in this period.

Drug susceptibility testing was done for the 1^st^ and 2^nd^ line drugs using recommended MICs by absolute concentration method [7].

**Table 1:** Spoligotypes of *M. tuberculosis* strains from Pskov area in Russia.

SIT*	Family	Number of strains	Spoligoprofile
1	Beijing	40	□□□□□□□□□□□□□□□□□□□□□□□□□□□□□□□□□□▪▪▪▪▪▪▪▪▪
35	Ural	2	▪▪▪▪▪▪▪▪▪▪▪▪□▪▪▪▪▪▪▪▪▪▪▪▪▪▪▪□□□▪□□□□▪▪▪▪▪▪▪
42	LAM	2	▪▪▪▪▪▪▪▪▪▪▪▪▪▪▪▪▪▪▪▪□□□□▪▪▪▪▪▪▪▪□□□□▪▪▪▪▪▪▪
46	Haarlem	2	▪▪▪▪▪▪▪▪▪▪▪▪▪▪▪▪▪▪▪▪▪▪▪▪□□□□□□□□□□□□□□□□□□□
47	Haarlem	2	▪▪▪▪▪▪▪▪▪▪▪▪▪▪▪▪▪▪▪▪▪▪▪▪▪□□□□□□▪□□□□▪▪▪▪▪▪▪
50	Haarlem	6	▪▪▪▪▪▪▪▪▪▪▪▪▪▪▪▪▪▪▪▪▪▪▪▪▪▪▪▪▪▪□▪□□□□▪▪▪▪▪▪▪
52	T2	1	▪▪▪▪▪▪▪▪▪▪▪▪▪▪▪▪▪▪▪▪▪▪▪▪▪▪▪▪▪▪▪▪□□□□▪▪▪□▪▪▪
53	T1	8	▪▪▪▪▪▪▪▪▪▪▪▪▪▪▪▪▪▪▪▪▪▪▪▪▪▪▪▪▪▪▪▪□□□□▪▪▪▪▪▪▪
102	T	3	▪▪▪▪▪▪▪▪▪▪▪▪□□□□▪▪▪▪▪▪▪▪▪▪▪▪▪▪▪▪□□□□▪▪▪▪▪▪▪
252	LAM	8	▪▪▪▪▪▪▪▪▪▪□□▪▪▪▪▪▪▪▪□□□□▪▪▪▪▪▪▪▪□□□□▪▪▪▪▪▪▪
254	LAM	3	▪▪▪▪▪▪▪▪▪▪▪▪▪▪□□□□□□□□□□▪▪▪▪▪▪▪▪□□□□▪▪▪▪▪▪▪
262	Ural	3	▪▪▪▪▪▪▪□□▪▪▪▪▪▪▪▪▪▪▪▪▪▪▪▪▪▪▪□□□▪□□□□▪▪▪▪▪▪▪
266	LAM	2	▪▪▪▪▪▪▪□▪▪▪▪▪□□□□□□□□□□□□▪▪▪▪▪▪▪□□□□▪▪▪▪▪▪▪
267	LAM	1	▪▪▪▪▪▪▪▪▪▪▪▪□□□□□□□□□□□□□▪▪▪▪▪▪▪□□□□▪▪▪▪▪▪▪
444	LAM	2	▪▪▪▪▪▪▪▪▪▪▪▪▪□□□□□□□□□□□□□▪▪▪▪▪▪□□□□▪▪▪▪▪▪▪
1288	Manu2	1	▪▪▪▪▪▪▪▪▪▪□□▪▪▪▪▪▪▪▪□□□□▪▪▪▪▪▪▪▪□□▪▪▪▪▪▪▪▪▪
2021	T	1	▪▪▪▪▪▪▪▪▪▪▪▪▪▪▪▪▪▪▪▪▪▪▪▪▪▪▪▪▪▪□□□□□□□□□□▪▪▪
3108	unknown	2	▪▪▪▪▪▪▪▪▪▪▪▪▪▪▪▪▪▪▪▪▪▪▪▪□□□□□□□□□□□□▪▪▪▪□▪▪
‘new’	unknown	1	▪▪▪▪▪▪▪▪□□▪▪□▪▪▪▪▪▪▪▪▪▪▪▪▪▪▪▪▪▪□□□□□▪▪▪▪▪▪▪

* according to SITVITWEB [10].

**Figure 1:**
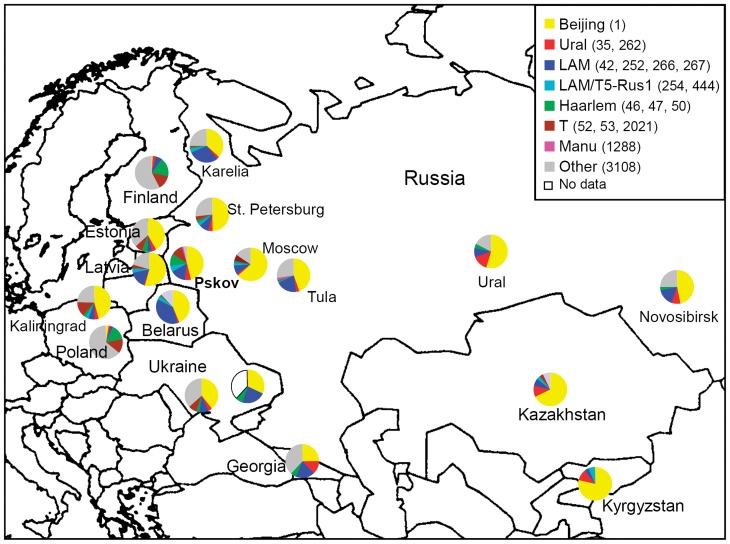
Geographic distribution of the main spoligotypes and genetic families identified in *M tuberculosis* strains in Pskov region and other areas in Russia, former Soviet Union and northern Europe.

**Figure 2:**
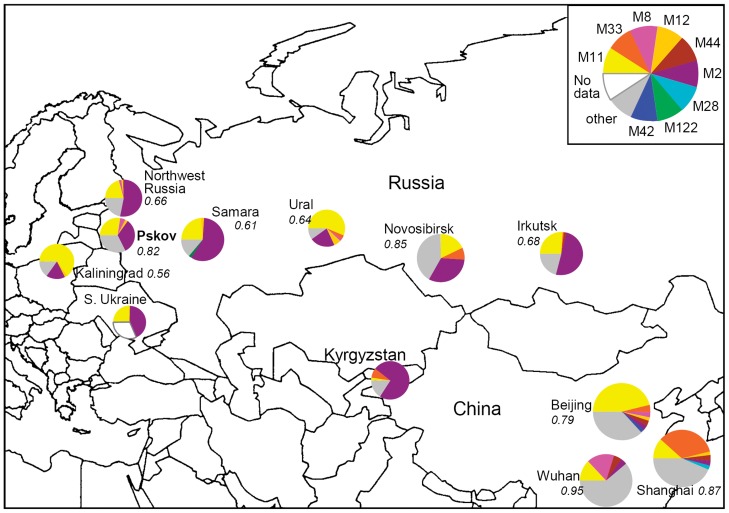
Geographic distribution of the main MIRU types of the Beijing genotypes identified in the Pskov region and other areas of the former Soviet Union and Asia.

**Figure 3:**
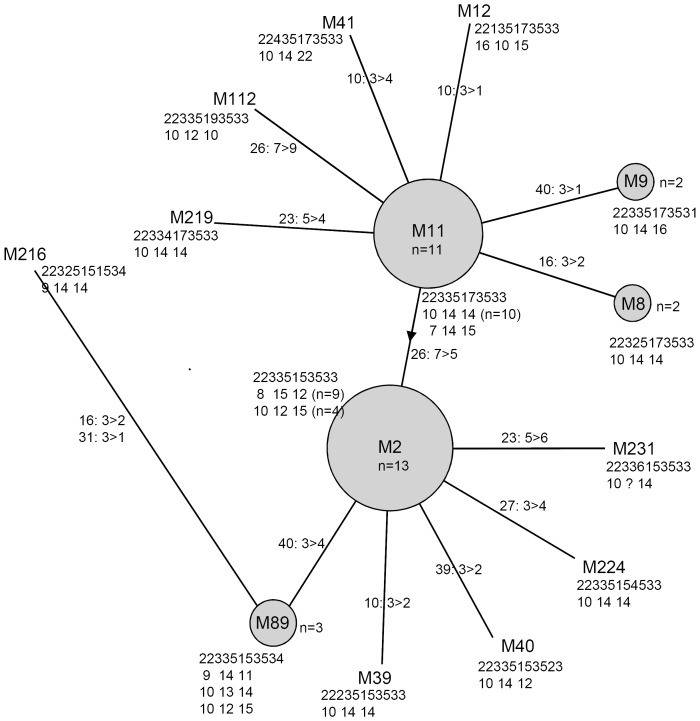
Minimum spanning tree of the 12-MIRU-VNTR profiles of the Beijing strains from the Pskov region in the northwestern Russia. 12-loci digital profile is shown in the 1^st^ line at the each node, alleles of 3 HV loci (VNTR-4120, VNTR-3820, QUB-3232) are shown in the 2nd line. Locus number and allele change are shown on branches. Circle size is roughly proportional to the number of strains.

### Genotyping

DNA was extracted using the recommended method [8]. Spoligotyping of isolates was performed as described by Kamerbeek et al. [9]. The spoligoprofiles were entered into Excel spreadsheets and compared with SITVITWEB, an international spoligotype database at Institut Pasteur de Guadeloupe [10].

VNTR (12 MIRU and 3 HV loci) analysis was performed as previously described [11], [12]. For allele calling of the HV loci we used a correspondence table kindly provided by Dr. T. Iwamoto.

The MIRU profiles were compared with MIRU-VNTRplus online database [13] in order to verify the lineage determination based on spoligoptying data. In addition, the MIRU profiles of the Beijing genotype strains were compared to the proprietary MIRU-VNTR database of the Beijing genotype [14], [15]; at the time of comparison this database included ∼2400 strains/profiles from all continents.

Analysis of the specific IS*6110* insertion characteristic of the LAM genetic family was done using multiplex PCR as described previously [16].

**Table 2:** Global distribution of the MIRU profiles identified in *M. tuberculosis* Beijing genotype strains from Pskov.

Type, % in database (if >0.5%)*	12-MIRU-loci profile	Pskov, number (%)	Former Soviet Union, area and %	Mainland China, area and %	East Asia other than China, country and %
M2 (14.8)	223325153533	13 (32.5)	Northwest 53, Samara 58, Kaliningrad 17, Ural 24, Irkutsk 50, South Ukraine 42, Kyrgyzstan 73	Beijing 2.7, Shanghai 2.7, Wuhan 3.6, Henan 6.2,	Japan 0.5
M11 (25.4)	223325173533	11 (27.5)	Northwest 21, Samara 24, Ural 56, Irkutsk 26, Kaliningrad 65, South Ukraine 26, Kyrgyzstan 2.6	Beijing 46, Shanghai 12, Wuhan 13, Henan 19	Japan 24, Laos 28, Mongolia 66, Vietnam 21
M8	22325173533	2 (5)	Northwest 2.1, Ural 2	Beijing 2.7, Wuhan 17, Henan 3.1	Japan 0.3
M40 (0.5)	223325153523	1 (2.5)	Kaliningrad 2.5, Irkutsk 3.5, Kyrgyzstan 2.6	Shanghai 1.3	
M9	22335173531	2 (5)	Northwest 2.1, Kaliningrad 2.5	Beijing 2.7, Shanghai 1.3, Henan 3.1	Mongolia 11, Vietnam 1.9
M12	22135173533	1 (2.5)	Northwest 2.1, Samara 1.6, Ural 6, Kaliningrad 2.5	Beijing 1.3, Shanghai 1.3, Henan 6.2	Japan 13
M39	22235153533	1 (2.5)			
M41	22435173533	1 (2.5)	Kaliningrad 5		Japan 0.3, Vietnam 1.9
M89	22335153534	3 (7.5)	Ural 2, Irkutsk 0.9		
M112	22335193533	1 (2.5)		Beijing 1.3	
M216	22325151534	1 (2.5)			
M219	22334173533	1 (2.5)			Japan 0.3
M224	22335154533	1 (2.5)			
M231	22336153533	1 (2.5)			

* according to the updated version (2400 strains) of the MIRU global database of Beijing genotype [14], [15] updated with more recent raw data on Henan, Mongolia, Japan-Osaka [44], Laos [45], Ukraine [32], Kaliningrad [30], Kyrgyzstan [21].

### Resistance mutations detection

Mutations in *katG315* and *inhA* promoter region and *ahpC* associated with INH resistance and mutations in *rpoB* rifampin resistance determining region (RRDR, codons 507–533) associated with RIF resistance were detected by using TB-Biochip kit (Biochip-IMB, Moscow, Russia) following the manufacturer's instructions.

### Phylogenetic and statistical analysis

Hunter Gaston index (HGI) was calculated as described previously [17] and was used to evaluate discriminatory power of the typing schemes.

PARS program of the PHYLIP 3.6 package [18] was used to reconstruct the minimum spanning tree of the VNTR digital profiles treated as categorical variables.

**Table 3:** Discriminatory capacity of different VNTR typing schemes applied to the Beijing genotype strains from the Pskov region in the northwestern Russia.

Characteristic	12-MIRU loci	+3 HV*	+3232	+3820	+4120	+3232 and 3820	+3232 and 4120	+3820 and 4120
Cluster** range	2–13	2–10	2–10	2–11	2–10	2–10	2–10	2–10
Number of clusters	5	5	5	5	6	5	5	5
Clustered	31	26	27	27	29	26	27	26
Singletons	9	14	13	13	11	14	13	14
HGI	0.823	0.896	0.886	0.873	0.885	0.896	0.886	0.896

* Three hypervariable (HV) loci, namely, QUB3232, VNTR3820, VNTR4120.

** Cluster is defined as a group of strains with identical multi-locus digital signature.

**Table 4:** Drug susceptibility and prevalence of drug resistance mutations in *M. tuberculosis* strains of Beijing, LAM and other genotypes isolated from TB patients in Pskov region of Russia.

Phenotype*, genotype	All strains, n = 90	Beijing genotype, n = 40	LAM genotype, n = 18	Other genotypes, n = 32	Beijing M2, n = 13	Beijing M11, n = 11
Fully susceptible	34	5	5	24	0	0
RIF-resistant	43	26	10	7	12	9
INH-resistant	50	30	13	7	13	10
STR-resistant	55	34	13	8	13	11
EMB-resistant	33	17	10	6	10	4
PZA-resistant	6	3	2	1	2	1
OFL-resistant	6	4	2	-	1	1
ETH-resistant	12	3	8	1	1	-
KAN-resistant	16	5	7	4	3	1
CAP-resistant	11	2	6	3	-	-
MDR	42	25	10	7	12	9
XDR	5	3	2	-	1	-
*rpoB531*	24	19	-	5	10	6
*rpoB526*	2	1	1	-	1	1
*rpoB516*	8	-	8	-	-	-
*rpoB507*	1	-	-	1	-	-
*rpoB511*	1	-	-	1	-	-
*rpoB512*	2	1	1	-	-	-
*rpoB* wild type	52	19	8	25	2	4
*katG* 315-ACC	44	26	12	6	12	8
*inhA* -15T	10	-	10	-	-	-
*katG* 315-ACC & *inhA* −15 T	10	-	10	-	-	-

* RIF, rifampin; INH, isoniazid; STR, streptomycin; EMB, ethambutol; PZA, pyrazinamide; OFL, ofloxacin; ETH, ethionamide; KAN, kanamycin; CAP, capreomycin; MDR, multidrug-resistant; XDR, extremely drug resistant.

A 2×2 χ^2^ test was used to detect any significant difference between the two groups. Yates corrected χ^2^ and *p*-values were calculated with 95% confidence interval using EpiCalc 2000 version 1.02 software [19].

## Results and Discussion

### Study sample and population structure

The studied collection included 90 *M. tuberculosis* strains isolated from permanent residents in the Pskov oblast, northwestern Russia, in 2008–2009. No preliminary selection of strains based on their drug resistance or patient status was made. These strains were isolated from patients without proven epidemiological links based on standard contact investigation, 59 males and 31 females, aged from 20 to 73 years old and admitted to the clinics of the Pskov oblast anti-tuberculosis dispensary in 2008–2009. Most of them had infiltrative-pneumonic lung TB (71.1%) and disseminated lung TB (18.8%), according to Russian classification of diseases (both fall within international definition ‘Tuberculosis of lung, confirmed by sputum microscopy with or without culture’, http://apps.who.int/classifications/icd10/browse/2010/en#/A15.0). Sixty-five (72.2%) patients were newly diagnosed patients who never received anti-TB treatment while 25 (27.8%) patients were previously treated. All enrolled patients were HIV-negative, some of them were former prison inmates (n = 14) and alcoholic (n = 12). These two latter subgroups did not overlap: only one patient was both ex-prisoner and alcoholic. As mentioned above the strains were selected randomly for this study and the high rate of former prison inmates (14/90 [15.5%]) reflect the real situation in the country. In North-West Federal District of Russian Federation (including Pskov oblast), the TB incidence among prisoners was 957/100,000 in 2010. In Russia as a whole, the rate of prisoners among newly-diagnosed TB patients was reported to be 12% in 2009 (T. Otten, personal communication).

**Table 5:** Drug susceptibility of *M. tuberculosis* strains of different genotypes from newly-diagnosed and previously-treated patients.

Characteristic	Newly-diagnosed patients (n = 65), number, %	Drug-resistant strains of the respective genotype, number, %	Previously-treated patients (n = 25), number, %
Genotype			
Beijing	29, 44.6	23, 79.3	11, 44.0
LAM	9, 13.8	4, 44.4	9, 36.0
Haarlem	7, 10.8	1, 14.3	2, 8.0
T	12, 18.5	1, 8.3	1, 4.0
Ural	4, 6.2	2, 50.0	1, 4.0
Phenotype			
Drug-resistant	31, 47.7		24, 96.0
Drug-susceptible	34, 52.3		1, 4.0

Spoligotyping was applied to all strains as a primary typing tool to define the major lineages shaping the general population structure of *M. tuberculosis* in the Pskov region. Ninety strains were subdivided into 18 spoligotypes. Following comparison with SpolDB4 database they were assigned to the SIT numbers and genetic families (Table 1). In addition, the strains were compared to the MIRU-VNTRplus database and also subjected to the LAM-specific PCR which helped us to clarify/change the family status of some spoligotypes. In particular, SIT254, SIT444 (T5_RUS1) SIT266, SIT267 (T family) were redefined as LAM; SIT35 and SIT262 (Haarlem4) were redefined as Ural. Consequently, this changed a relative proportion of strains belonging to certain genetic families in our collection. In total, the Beijing genotype (40/90) was found the most prevalent in our setting followed by LAM (n = 18), T superfamily (n = 13), Haarlem (n = 10), Ural (n = 5), and Manu2 (n = 1). The family status remained unknown for 3 isolates after comparison with SITVITWEB international database [10].

The history of alcoholism and imprisonment were not significantly associated with infection with particular strain genotype in this study. LAM family was the most prevalent among alcoholics (n = 5) followed by Beijing (n = 3), T (n = 3) and Haarlem (n = 1). Interestingly, all 3 Beijing strains and only 1 of 5 LAM strains were from newly-diagnosed patients. On the other hand, all three Beijing strains and 4 of 5 LAM strains were drug resistant.

The 14 strains isolated from ex-prisoners belonged to the following families: Beijing (n = 7), Haarlem (n = 3), LAM (n = 1), T (n = 1), Ural (n = 1), unknown (n = 1). When contrasting ex-prisoners and patients without prison history, the Beijing genotype constituted similar proportions in both groups: 7 (50.0%) of 14 and 34 (44.7%) of 76, respectively. In contrast, LAM strains were isolated from 1 (7.1%) of 14 ex-prisoners and 17 (22.4%) of 76 other patients, respectively (*P* = 0.3). Regarding drug resistance of ‘prison’ strains, all 7 Beijing strains and only 1 of 7 non-Beijing strains were drug-resistant. Previous studies in the former Soviet Union found a higher rate of Beijing genotype in ex-prisoners [20], [21], [22]. An exception was Central Russian study that highlighted an important prevalence of the LAM family (44.8%) similar to that of the Beijing genotype (43.7%) [23]. Summing up, a better transmission of Beijing strains under overcrowded conditions in a prison environment has been demonstrated in most settings but not in our study.

To view the distribution of the families identified here beyond its target area, we compared the spoligotype based population structures of *M. tuberculosis* in Pskov region and other areas of the former USSR as well as Poland and Finland [2], [3], [14], [20], [21], [24], [25], [26], [27], [28], [29], [30], [31], [32], [33], [34], [35], [36], [37]; the pie charts are shown in Fig. 1. Its is clear and otherwise well-known that Beijing genotype is relatively or absolutely prevalent throughout all countries of the former Soviet Union but found in extremely low rate in Finland and Poland. Sinkov et al. [38] have recently suggested an intriguing hypothesis about primary penetration of the Beijing genotype from China to the Soviet Union only in the 20^th^ century but not earlier. They reasoned that Finland was a part of the Russian Empire and since Beijing is not endemic in Finland, apparently it should have arrived to Russia after Finland's independence in 1918. However one should keep in mind that although TB mortality was very high in Russia early 20^th^ century (∼400/100,000 [39]), the country remained agrarian and not densely populated. Finland became a part of the Russian Empire only in 1810, furthermore it kept its semi-autonomous status and the Russian presence was limited to the civil servants and military garrisons and constituted only few percent of the total population of Finland (http://en.wikipedia.org/wiki/Russians_in_Finland). In this view, the Beijing strain would hardly be disseminated by Russians throughout Finland in any case.

Regarding countries of the former Soviet Union, in spite of the very high rate of the Beijing genotype in all settings, one may note some gradient of its prevalence with higher rate in Central Asia (Kazakhstan and Kyrgyzstan, >60%) versus relatively lower rate (mostly 40% or less) in the western borderline areas of the former USSR from Georgia in south to Pskov and Karelia in north (e.g., *P* = 0.01 for Pskov versus Kazakhstan comparison, Fig. 1).

The 2^nd^ important family in this study was LAM (20%). This family is also found in other areas of the European part of the former Soviet Union, e.g. Kharkov in South Ukraine (23% [26]), Kaliningrad (18% [30]), but in lesser rate in Ural and west Siberia (9–10% [31], [40]) although a more recent study in Novosibirsk in west Siberia described 18% of LAM [25]. In spite of the phylogeographically meaningful name (Latin American Mediterranean), at present this family is globally distributed and its within-Russian gradient may be observed only on the large scale when comparing European versus Asian parts of Russia and former Soviet Union as a whole.

A feature specific for Pskov is a relatively higher rate of Haarlem and T types which is similar to Poland and Finland but quite different from other Russian regions. This is well in line with westernmost location of Pskov region within mainland Russia.

It is also interesting to note some gradient of the less known genetic family Ural in the south-eastern area on the map. It was recently suggested that central Eurasia may be an area of primary dissemination of this family, north/north-east Pontic area would be its origin [41] while more distant areas of northwestern (including Pskov) and central Russia exhibit a negligible rate of the Ural family (Fig. 1).

### Structure of the Beijing genotype subpopulation

We further analysed the population structure of the Beijing family that was predominant in this study and is known to be epidemiologically and clinically important genotype of *M. tuberculosis* [10], [15], [22], [42], [43], [44], [45]. To this end, we used 12-MIRU-VNTR typing followed by comparison with international database of the Beijing genotype [14], [15]. We did not aim to achieve a high discrimination of single strains but rather to identify the major MIRU-types and compare their distribution in Pskov region against neighboring and more distant areas in Eurasia.

The 12-MIRU typing differentiated 40 Beijing strains into 14 types while two types M2 (223325153533) and M11 (223325173533) were found in similar and high rates (Table 2); for reference and comparability purposes it may be noted that these types correspond to MIT16 and MIT17, respectively, in SITVIT database in Pasteur Institute of Guadeloupe [10]. These two types are major types of Beijing genotype on the global scale (M2 prevalent throughout Russia and ex-USSR and M11 prevalent in Russia and East Asia) and also account for most of Beijing strains in Russia although in different proportions across its regions. The comparison of prevalence of the Beijing MIRU types in this study and elsewhere is shown in Table 2 and Fig. 2. The major Russian type M2 is also found in Pskov although in smaller rate. In the article of Drobniewski et al. [22] it was suggested that M2 is more prevalent in prison population while M11 is more prevalent in civilians. This would imply more capacity to disseminate of M2 versus M11. Prison-based studies in Kyrgyzstan [21] and Georgia [20] found very high rate of Beijing/M2 suggesting a particular capacity of this Beijing subtype to spread in a prison ( = overcrowding) environment. On the other hand, these findings were not replicated in the Russian study in the Moscow setting [24]. Thus the relation of the M2 or M11 MIRU-types to the increased transmissibility remains uncertain. In the 12-MIRU tree the central position of M2 and M11 types (Fig. 3) correlates with the same star-like topography of the global MIRU-based tree of the Beijing genotype [14] confirming ancestral position of these types in Russia.

Other Beijing MIRU types found in our setting were shared by one to three strains (Table 2, Fig. 3). Some of them (types M8, M9, M12, M40) were also reported in other areas of the former USSR as well as in East Asia, usually in not high percent. It was suggested that a low level of diversity of 12-MIRU loci (types) in Russia is linked to the recent dissemination of these strains across the country. Compared to the diversity of the Beijing populations in other Russian regions, the Beijing diversity (HGI value) in Pskov was somewhat higher (Fig. 2). This may be explained by the almost equal presence of the two major types M2 and M11.

The globally standardized VNTR format initially based on 12 ‘classical’ loci [11] was followed by the 24-loci scheme [46]. Although this scheme is generally accepted, in the recent years, the importance of development of the complementary region-adjusted schemes has been recognized [12], [47], [48], [49]. Our previous study of 27 VNTR loci in the Beijing strains mainly from St. Petersburg led us to suggest a critical use of 3 hypervariable loci for higher resolution VNTR typing of Beijing strains in Russia [48]. Twelve MIRU plus three HV loci approach was also used previously in the Beijing dominated setting (Kyrgyzstan) [21].

Here we tested these loci on the subset of Beijing strains from Pskov. Indeed their use somewhat improved a discrimination as is clear in the minimum spanning tree of the 12-MIRU loci with added 3-HV data (Table 3, Fig. 3). MIRU-12 identified 14 profiles, including 5 types shared by 2 to 13 strains, with HGI of 0.82. The use of 3 HV loci resulted in 18 profiles 5 types shared by 2 to 10 strains, HGI was 0.89. On the other hand, use of all three HV loci was apparently redundant (Table 3): two HV loci (VNTR-3820 and QUB-3232, or VNTR-3820 and VNTR-4120) would be sufficient to complement the 12-MIRU typing without loss of discrimination.

### Drug resistance properties: genotype and phenotype

The drug susceptibility testing of the 1^st^ and 2^nd^ line anti-TB drugs and detection of the genetic determinants of drug resistance to the major drugs rifampin (RIF) and isoniazid (INH) was done for all strains. The results are shown in Table 4. We also compared distribution of drug resistance in major genetic families Beijing (and its subtypes M2 and M11) and LAM.

Thirty-four strains were susceptible while 56 strains were found resistant to at least one drug; of these latter 42 strains were MDR and 5 strains were XDR. Regarding strains from newly-diagnosed patients, 34 were susceptible, 31 drug-resistant, 18 MDR, 1 XDR. The level of monoresistance generally reflects the quality of treatment and compliance. In this study, five strains were resistant to a single drug (streptomycin [STR]), while 4 of them were Beijing genotype and one strain belonged to T family (SIT53). STR is known to have been widely used in the 1990s in Russia and almost all Russian drug resistant strains regardless of their genotype are resistant to at least STR. Thus this finding of STR monresistant strains is not surprising. However absence of RIF and INH monoresistant strains may be considered as an indirect evidence of sufficient level of compliance of patients infected with drug susceptible strains in Pskov.

It should be noted that both major families Beijing and LAM had similar rate of MDR strains: 25 of 40 Beijing versus 10 of 18 LAM strains were MDR (*P* = 0.83). On the other hand, both included a higher percent of MDR strains when compared to other genotypes pooled together. Twenty-five of 40 Beijing versus 7 of 32 other (non-Beijing, non-LAM) strains (*P* = 0.001 5.95 [2.07; 17.09], and 10 of 18 LAM versus 7 of 32 other (non-Beijing, non-LAM) strains (*P* = 0.03, OR 0.54 95% CI [0.30; 0.99]) were MDR. A recent Ukrainian study showed an association of MDR with LAM but not Beijing genotype [26], i.e. 11 of 16 LAM and 17 of 31 Beijing were MDR. Even if a small sample size may be a reason, this result suggests (as in our study) a general trend of the family/phenotype association in a high TB-burden area: the drug resistance is ultimately associated with all major circulating families. In case of Russia these are Beijing and LAM. This finding highlights an importance to consider not only the notorious Beijing genotype but also other circulating families in an area, in particular, to compare not only the Beijing genotype versus all other families taken together but perform more detailed comparisons.

On the other hand, one should note an interesting distribution of the particular mutations/codons in *rpoB* in different families. For example, *rpoB531* mutations were more frequently found in Beijing strains while LAM RIF resistant strains usually had *rpoB516* mutations. A similar finding of the *rpoB516* mutations found mainly in LAM strains was published by Ignatova et al. [23]. Different mechanisms related to the second order selection [50] and/or compensatory mutations in other genes [51] may be hypothetically related to this bias. On the other hand, the quality of the drug used in different countries may also hypothetically contribute to the preferential selection of some mutations.

An association of *rpoB531* mutation with Beijing genotype was shown in many other studies carried out in the former Soviet Union [22], [24], [25], [30], [32]. On the other hand, the very high rate of this (otherwise relatively most frequent) mutation in Beijing strains is not inevitable feature of this genotype only. For example, a study in Bulgaria found high rate of this mutation in the RIF-resistant strains of different (non-Beijing) genotypes [52].

The *katG315* AGC>ACC mutation was frequently described in INH resistant strains, especially in the high TB-burden areas [22], [25], [30], [32]. Thus its finding in the present study is not unexpected: *katG315* AGC>ACC was found in similar rate in 26 of 30 Beijing and 12 of 13 LAM, and 6 of 7 other genotypes' INH-resistant strains. An intriguing feature that is also observed in Table 4 is a clear difference in prevalence of *inhA* −15C>T promoter mutations. First, all 10 strains with −15C>T mutation harbored also *katG315* mutation; this is not unusual since these *inhA* mutations are known to provide secondary mechanism of INH resistance. Second, and more remarkable is that these 10 strains were all and only different types of the LAM family. One should note that this was not observed in the Moscow study where *inhA* promoter mutations were found in different genotypes [24].

In terms of resistance properties within Beijing subtypes (MIRU types), neither M2 nor M11 included fully susceptible strains, and almost completely consisted of MDR strains. Multidrug resistance was frequent among Beijing M2 and M11 types (87.5%) than in other Beijing MIRU types taken together (12.5%) (*P*<0.0001). Interestingly all drug susceptible Beijing strains belonged to the minor types M8, M39, M41, M89, M219 и M231.

We looked more closely at the subsample of our collection representing strains isolated form newly-diagnosed patients (Table 5). The Beijing strains were found in similar rate in both newly-diagnosed and previously-treated TB subsamples (44.6 and 44.0%, respectively). In contrast, LAM strains were more visible among previously-treated patients (36.0 vs 13.8%, *P* = 0.04 OR 3.50 95% CI [1.19; 10.29]). Taken together with a high rate of drug resistance among Beijing strains from new TB cases, these findings suggest the critical impact of the Beijing genotype on the current situation with TB in this region in northwestern Russia.

### Concluding remarks

The *M. tuberculosis* population in Pskov region in northwestern Russia features all major genetic families characteristic for Russia and its European part, the Beijing and LAM genotypes being the most prevalent. In contrast to other Russian regions, *M. tuberculosis* population in Pskov is marked with a relatively higher proportion of Haarlem and T types.

The 12-MIRU typing differentiated 40 Beijing strains into 14 types while two largest types were M2 (223325153533) prevalent throughout Russia and ex-USSR and M11 (223325173533) prevalent in Russia and East Asia. Whereas 12-MIRU identified 14 profiles (HGI = 0.82), the use of 3 HV loci improved discrimination resulting in 18 profiles (HGI = 0.89).

Comparison with drug resistance data suggests a general trend of the family/phenotype association in a high TB-burden area: the multidrug resistance is associated with all major circulating families. In case of Russia these are Beijing and LAM. This highlights an importance to monitor not only the notorious Beijing genotype but also other *M. tuberculosis* families in an area.

The prevalence of the Beijing genotype along with high rate of drug resistance among Beijing strains from new TB cases suggest the critical impact of the Beijing genotype on the ongoing transmission of MDR-TB in this region in northwestern Russia.
